# Efficacy of intramuscular methyl prednisolone in preventing restenosis after coronary artery stenting with bare-metal stainless steel stent: a double-blind, randomised, controlled clinical trial

**DOI:** 10.5830/CVJA-2010-039

**Published:** 2011-04

**Authors:** M Namdari, M Ghafarzadeh, MA Nikoo

**Affiliations:** Department of Cardiology, Lorestan University of Medical Sciences, Khoramabad, Iran; Department of Medicine, Lorestan University of Medical Sciences, Khoramabad, Iran; Tehran University of Medical Sciences, Farzan Clinical Research Institute, Tehran, Iran

**Keywords:** percutaneous transluminal coronary angioplasty stenting, restenosis, prednisolone

## Abstract

**Abstract:**

The aim of this study was to compare the mid-term outcome of patients receiving intramuscular methyl prednisolone before and after the procedure of coronary artery stenting. The study was conducted during 2007 and 2008 and compared the two arms of the study for the rate of restenosis six months after stenting. The control arm (100 patients) received only the usual preventive measures but the glucocorticoid arm (100 patients) received two doses of intramuscular methyl prednisolone (40 mg) at two-week intervals, the first at the time of the procedure. They also received the usual preventive measures

There was no statistically significant difference between the two arms for the rate of restenosis. When separately analysing for three vessels and for gender, there was no statistically significant difference either.

Lowering the dose of corticosteroid would greatly reduce the efficacy for preventing restenosis after coronary artery stenting. Therefore, if we are to achieve acceptable effectiveness with intramuscular prednisolone, we should administer increased doses at shorter intervals, which could be the target of further studies. However, there would be more chance of side effects with increased frequency of dosing.

## Abstract

Although percutaneous transluminal coronary angioplasty stenting has greatly improved the outcome of patients with coronary artery disease,^1^–^3^ there have been reports of restenosis in as many as half the cases.^4^,^5^ Many prophylactic pharmacological interventions have been proposed to prevent restenosis after coronary artery stenting.^6^–^8^ Previous experimental and human studies have shown that inflammation plays a key role in the process of restenosis,^9^–^12^ and as glucocorticoids are one of the best known anti-inflammatory agents, theoretically, glucocorticoids should have beneficial preventive effects. Platelet function, smooth muscle cell proliferation and collagen synthesis as well as inflammatory cell migration and activation are some of the steps that are involved in the process of restenosis and are also targets of glucocrticoid action.^13^–^17^ Many studies have been conducted to evaluate the clinical efficacy of this treatment modality for avoiding restenosis, with variable and sometimes even opposing results.^18^-^23^

Weighing up the controversial results of these studies, it seems that the route of administration, dosage and duration of glucocrticoid therapy can affect the results achieved.^24^,^25^ There are three main routes for systemic administration of glucocorticoids; intravenous, intramuscular and the oral route. As the intramuscular route of administration is more convenient and with fewer complications than the intravenous route, and it does not have the problems of non-compliance that the oral route does, we conducted this double-blind, randomised, controlled trial to compare mid-term outcome of patients receiving intramuscular methyl prednisolone before and after the procedure of stenting with patients receiving only the usual preventive measures.

## Methods

This double-blind, randomised clinical trial was conducted during 2007 and 2008 in the Shahid Madani Heart Centre of Lorestan in Iran. Patients who were admitted to hospital for percutaneous coronary intervention with bare-metal stainless steel stents were enrolled in the study. Exclusion criteria were age below 40 years and having diabetes mellitus.

Two hundred patients were selected consecutively and were randomly assigned to two groups. The groups were matched with regard to age, gender and modifiable risk factors such as smoking, family history, hyperlipidaemia and hypertension. Forty-eight hours before angioplasty, one group of patients (glucocorticoid arm) received one dose (40 mg) of intramuscular methyl prednisolone. The other group (control arm) received nothing except the usual management, which the glucocorticoid arm also received.

Thereafter, all patients were admitted to the critical care unit (CCU). They were all well hydrated, had a chest X-ray, and underwent routine laboratory studies and a diagnostic angiographic study before the procedure of percutaneous coronary angioplasty. In our centre we use clopidogrel for 45 days prior to the procedure. Finally, percutaneous coronary angioplasty with stenting was performed on both groups. Patients were discharged 24 hours after the procedure. Fourteen days later, the patients in the glucocorticoid arm returned to our hospital to receive a second intramuscular dose (40 mg) of methyl prednisolone. Follow-up angiography was done six months after stenting.

Endpoints in our study were myocardial infarction, SCD, unstable angina, a positive stress echocardiographic test and observation of stenosis in the follow-up angiography. These endpoints meant that restenosis had occurred. All steps, that is, patient selection and randomisation, the initial studies, the first and second angiography, angioplasty and injections were blinded and only the head nurse of the CCU knew the patients.

Numerical variables are presented as means ± SD and categorised variables are summarised as absolute frequencies and percentages. Categorical variables were compared using the chi-square test or Fisher’s exact test if required. For statistical analysis, the statistical software SPSS version 13.0 for windows (SPSS Inc., Chicago, IL) was used. All *p*-values were two-tailed, and statistical significance was defined as *p* ≤ 0.05.

## Results

In this double-blinded, randomised clinical trial, 200 patients were included and they were divided into two groups of the same size. The mean diameter and length of stents was 2.7 mm and 19 mm, respectively. The patients were matched regarding age, gender and four modifiable risk factors: hypertension, hyperlipidaemia, smoking and family history. Characteristics of the two groups regarding age and gender are shown in Table 1.

**Table 1.** Characteristics And Rate Of Restenosis In The Two Arms

*Arm*	*n*	*Age (mean ± SD)*	*Female n (&percnt;)*	*Male n (&percnt;)*	*Restenosis n (&percnt;)*	*Without restenosis n (&percnt;)*
Glucocorticoid	100	60.29 ± 7.28	42 (42)	58 (58)	21 (21)	79 (79)
Control	100	60.44 ± 7.29	46 (46)	54 (54)	24 (24)	79 (79)

Twenty-one cases of restenosis were observed in the glucocorticoid arm of the study and 24 in the control arm. Restenosis was estimated with QCA. There was no statistically significant difference between the two arms in the rate of restenosis. With regard to the two genders and three vessels involved, we could not find any statistically significant difference between the two arms (Tables 2, 3).

**Table 2.** Characteristics Of The Two Arms For Each Gender

*Arm*	*Gender*	*Total n (&percnt;)*	*Without restenosis n (&percnt;)*	*With restenosis n (&percnt;)*	p*-value*
Glucocorticoid	Male	54 (100)	40 (74.1)	4 (25.9)	0.831
Control		58 (100)	44 (75.9)	14 (24.1)	
Total		112 (100)	84 (75)	28 (25)	
Glucocorticoid	Female	46 (100)	39 (84.8)	7 (15.2)	0.419
Control		42 (100)	32 (76.2)	10 (23.8)	
Total		88 (100)	71 (80.7)	17 (19.3)	

**Table 3.** Characteristics Of The Two Arms For Different Vessels

*Arm*	*Vessel*	*Total n (&percnt;)*	*Without stenosis n (&percnt;)*	*With stenosis n (&percnt;)*	p*-value*
Glucocorticoid	Left anterior descending	40 (100)	31 (77.5)	9 (22.5)	0.99
Control		50 (100)	38 (76)	12 (24)	
Glucocorticoid	Left circumflex	27 (100)	21 (77.8)	6 (23.1)	0.99
Control		27 (100)	21 (77.8)	6 (22.2)	
Glucocorticoid	Right coronary artery	23 (100)	17 (73.9)	6 (17.6)	0.517
Control		23 (100)	17 (73.9)	6 (26.1)	

## Discussion

Our results did not show a preventive role of intramuscular methyl prednisolone in decreasing the rate of restenosis after percutaneous stenting of coronary arteries. Also, there was no significant statistical difference in the subgroups of gender and vessel involved.

Despite the controversial results of previous studies regarding the efficacy of glucocorticoids in preventing restenosis, there is a widely accepted protocol that has been proved to be effective in most clinical trials performed with glucocorticoids.^18^–^22^ To understand the lack of efficacy of our protocol, one should compare the time–action profile of our study with this accepted protocol, which includes administration of oral prednisone for a total of 45 days in different doses: 1 mg/kg for the first 10 days, 0.5 mg/kg for the next 20 days and 0.25 mg/kg for the last 15 days, starting on the day of the procedure or the following day.

By comparison, our protocol includes administration of two intramuscular doses of 40 mg of methyl prednisolone; the first dose 24 hours before the procedure and the second 14 days afterwards. Oral prednisolone exerts its effect in one to two days and intramuscular methyl prednisolone exerts its effect in one to four weeks. As the potency of the drugs is equal and their bioavailability is almost equal, using our protocol, an 80-kg patient is exposed to 1/45 the amount given to the patients in the reported protocol.^26^

## Conclusion

As previous studies have shown,^25^ lowering the dose of cortico-steroids from this accepted protocol to even half the dose shows no efficacy in preventing restenosis after stenting, So if we are to achieve acceptable effectiveness for intramuscular prednisone, we should increase the doses using shorter intervals, which could be the target of further studies. However, there would be more chance of side effects with more frequent doses.
